# Cardiorespiratory fitness, body mass index, cardiovascular disease, and mortality in young men: A cohort study

**DOI:** 10.3389/fpubh.2023.1076065

**Published:** 2023-02-15

**Authors:** Alexander Wilhelm Gorny, Jonathan Yap, Jia Wei Neo, Wei En Chow, Khung Keong Yeo, Chuen Seng Tan, Falk Müller-Riemenschneider

**Affiliations:** ^1^Centre of Excellence for Soldier Performance, Singapore Armed Forces, Singapore, Singapore; ^2^Saw Swee Hock School of Public Health, National University of Singapore and National University Health System, Singapore, Singapore; ^3^National Heart Centre Singapore, Singapore, Singapore; ^4^Duke-NUS Medical School, National University of Singapore, Singapore, Singapore; ^5^Department of Cardiology, Changi General Hospital, Singapore, Singapore; ^6^Yong Loo Lin School of Medicine, National University of Singapore, Singapore, Singapore; ^7^Digital Health Center, Berlin Institute of Health, Berlin, Germany

**Keywords:** cardiorespiratory fitness, body mass index, Asian, young men, major acute cardiovascular event, all-cause mortality

## Abstract

**Objective:**

We examined the association between cardiorespiratory fitness (CRF), body mass index (BMI), incidence of major acute cardiovascular events (MACE), and all-cause mortality (ACM).

**Methods:**

We conducted a retrospective cohort study involving 212,631 healthy young men aged 16 to 25 years who had undergone medical examination and fitness testing (2.4 km run) from 1995 to 2015. Information on the outcomes of major acute cardiovascular events (MACE) and all-cause mortality (ACM) were obtained from the national registry data.

**Results:**

During 2,043,278 person-years of follow-up, 371 first MACE and 243 ACM events were recorded. Compared against the first run-time quintile, adjusted hazard ratios (HR) for MACE in the second to fifth quintiles were 1.26 (95% CI 0.84–1.91), 1.60 (95% CI 1.09–2.35), 1.60 (95% CI 1.10–2.33), and 1.58 (95% CI 1.09–2.30). Compared against the “acceptable risk” BMI category, the adjusted HRs for MACE in the “underweight,” “increased risk,” and “high-risk” categories were 0.97 (95% CI 0.69–1.37), 1.71 (95% CI 1.33–2.21), and 3.51 (95% CI 2.61–4.72), respectively. The adjusted HRs for ACM were increased in participants from the fifth run-time quintile in the “underweight” and “high-risk” BMI categories. The combined associations of CRF and BMI with MACE showed elevated hazard in the “BMI≥23-fit” category, which was more pronounced in the “BMI≥23-unfit” category. The hazards for ACM were elevated across the “BMI<23-unfit,” “BMI≥23-fit,” and “BMI≥23-unfit” categories.

**Conclusion:**

Lower CRF and elevated BMI were associated with increased hazards of MACE and ACM. A higher CRF did not fully compensate for elevated BMI in the combined models. CRF and BMI remain important targets for public health intervention in young men.

## Introduction

Physical activity (1) (PA), cardiorespiratory fitness (2–4) (CRF), and high body mass index (BMI) are powerful determinants of cardiovascular disease (CVD) and all-cause mortality (ACM) risks (5–7). Globally, less than one-quarter of male adolescents are sufficiently active (8), and this proportion declines further in adulthood (9). Having a high BMI is also an increasing concern in East and Southeast Asia, where the increase in obesity prevalence has been particularly rapid in young men (10).

Prospective studies have shown that BMI measured in adolescence and early adulthood predicted subsequent cardiovascular (CV) morbidity and mortality (11, 12). Our knowledge about CRF measured in adolescence and early adulthood has largely been based on cross-sectional studies examining the associations between low CRF and biomarkers of cardiometabolic risk (13, 14). Two recent systematic reviews of longitudinal studies have suggested that high CRF in adolescence could mitigate the adverse cardiometabolic effects of high BMI (15, 16).

There is consistent evidence to show that, among older adults, high CRF either attenuates or entirely mitigates the harmful effects of BMI in what is described as the “fat-but-fit” paradigm (17, 18). Evidence on whether the paradigm might extend to adolescents and young adults is sparse (19, 20). Therefore, to address this gap in the literature, we sought to study the independent and combined associations of CRF and BMI with CVD and ACM risk in healthy young men.

## Methods

### Study sample

**Figure 1:**
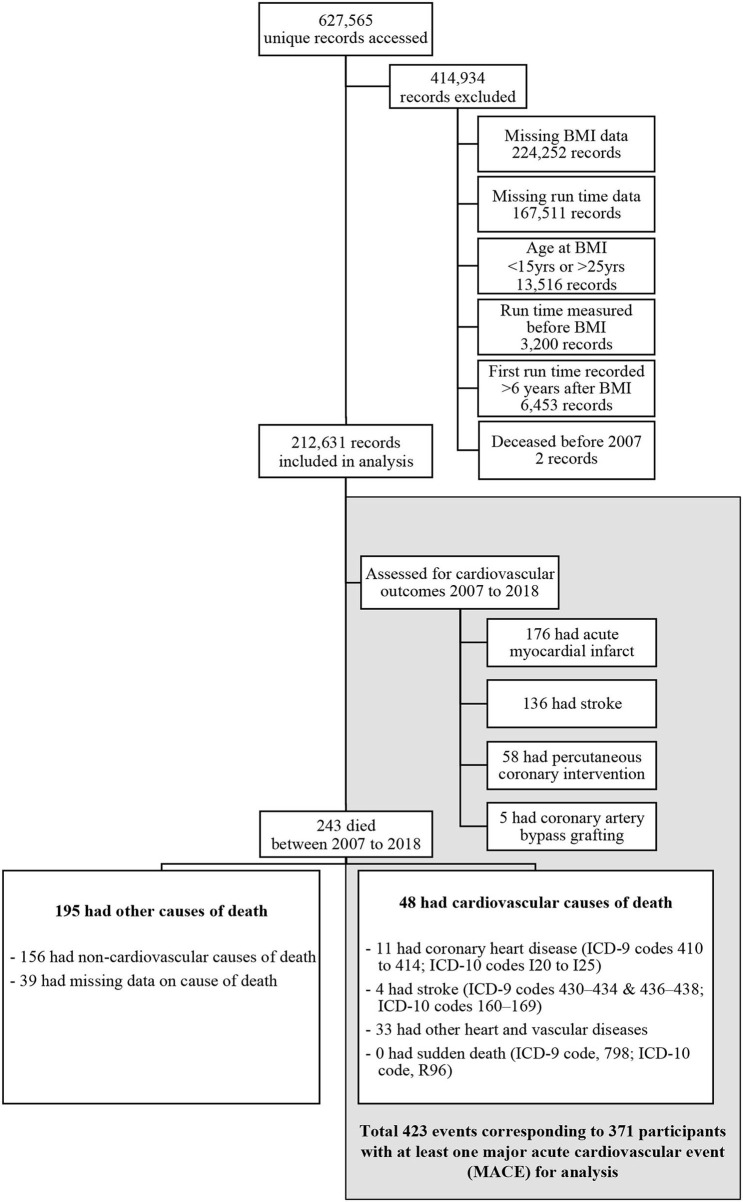
Study sample and outcome events.

At the age of 16 years, all male citizens and permanent residents of Singapore are required to enlist in the National Service with the majority entering the military. We accessed the electronic training and medical records of the Singapore Armed Forces to obtain the fitness and BMI data of all-male military service personnel (Figure 1). The participants were included in the study if they had had at least one BMI measurement and fitness test recorded in the years 1998–2015. The participants were subsequently excluded if their age at the time of earliest BMI measurement was below 16 or above 25 years. Subsequently, those whose first fitness test results had been conducted 6 years after their first BMI measurement were also excluded. Finally, any of the participants who were deceased before 01 January 2007 were removed from the analytical dataset.

Of the 627,565 unique male participants identified through the two databases, we excluded 224,252 potential participants due to missing BMI data and another 167,511 participants due to missing fitness test information. We excluded 13,516 participants based on our age criterion for BMI and another 3,200 whose first fitness test result was recorded before the first BMI measure. Another 6,453 participants were excluded as their first fitness test had been conducted more than 6 years after the first BMI measurement. Finally, we removed two participants who were deceased before 01 January 2007.

### Baseline measurements

Pre-enlistment medical examinations comprised a review of previous medical records, height taken barefoot on a stadiometer, weight measurements taken in underwear on an electronic scale, physical examination, a resting electrocardiogram, a dipstick urine test, blood tests, and a chest X-ray. As a rule, personnel assessed fit by a medical doctor were required to undergo annual physical fitness testing during National Service. For the purposes of this study, the earliest weight and height recorded were used to calculate BMI at baseline. The annual physical fitness test comprised multiple tests of which one was the 2.4 km timed run, also known as the modified Cooper's test (21). The fitness test was typically conducted during basic military training one to three years after the pre-enlistment medical examination. During the test, the participants were required to run six laps on a 400-m running track at the fastest speed possible. The run-times were recorded by a fitness instructor and entered into an electronic database. For this study, the earliest recorded 2.4 km run-time was used as the baseline measure.

### Outcome events

The primary outcome of our study was the time to the first major acute cardiovascular event (MACE) defined as acute myocardial infarction (AMI), stroke, coronary revascularization, or CV mortality, whichever had been recorded earlier. Using the national myocardial infarction (22), stroke (23), and cardiac reperfusion (24) registries, we identified all CV events registered from 01 January 2007, the start date of the AMI and stroke registries, until 31 December 2018. Using the national death registry data, we identified all deaths due to coronary heart disease (ICD-9 codes 410 to 414; ICD-10 codes I20 to I25), stroke (ICD-9 codes 430–434 and 436–438; ICD-10 codes 160–169), and sudden death (ICD-9 code 798; ICD-10 code R96). Deaths due to other heart and vascular diseases were identified manually by a physician on the research team to account for all deaths due to CV causes during the same period. The secondary outcome of our study was the time to death of any cause. An ACM event was defined as any death recorded in the national death registry occurring from 01 January 2007 until 31 December 2018.

### Study variables

The common identifier for all data points was Singapore's national registration identity card number. Data were collated from the respective databases and joined by data administrators in the National Registry of Diseases Office (NRDO) to maintain strict confidentiality. As event dates were coded according to month and year by the respective registries, we standardized the event dates to reflect the 15th day of the respective month. All statistical analyses were performed on the final deidentified dataset on a stand-alone terminal at the NRDO's data laboratory.

### Statistical analyses

Run-times were converted into seconds before determining the run-time quintile among the participants of the final study sample. The BMI data were categorized according to the World Health Organization's (WHO) public health action cutoff points for Asian populations (25) that defined the following categories: “underweight” for BMI<18.5 kg/m^2^, “acceptable risk” for BMI 18.5 to 22.9 kg/m^2^, “increased risk” for BMI 23.0 to 27.4 kg/m^2^, and “high risk” for BMI ≥27.5 kg/m^2^. Frequencies and percentages were calculated for each run-time quintile and BMI category along with the population mean and standard deviation (SD) of continuous variables. The numbers of the first MACE, the first ACM, the first AMI event, and the first stroke events were compiled for each run-time quintile and BMI category.

Either 01 January 2007 or the date of the first fitness test, whichever was later, was designated as the time of entry into the study period. The onset of the first MACE was specified as the event of interest in primary analyses. Censoring events comprised: Non-CV cause of death, missing causes of death, or being alive at the end of the study period (i.e., 31 December 2018) without the occurrence of MACE during follow-up. The onset of ACM was specified as the event of interest in secondary analyses with the censoring event defined as being alive at the end of the study period regardless of prior MACE. Follow-up time was defined as the time duration from entry to censoring events or the event of interest. Hazard ratios (HR) were estimated using Cox proportional hazard models. The adequacy of the proportional hazard assumptions was assessed using the global goodness-of-fit test proposed by Schoenfeld (26). Run-time quintile and BMI category were the two main exposures of interest. Both the first run-time quintile and the “acceptable risk” BMI category served as references. The basic adjusted models included the year of BMI measurement and the age at the time of entry into the study as confounders. The final models were adjusted for continuous measures of BMI and run-time, where appropriate. To test for linear trends across quintiles (or BMI categories), we assigned numerical values representing the median values of run-time (or BMI) for each run-time quintile (or BMI category). When a test for linear trend returned a non-significant result, we assessed whether the exposure as a categorical variable was a significant factor, meaning at least one of the non-reference HRs was not a significant factor for the model in question. Finally, adapting the approach of Henriksson and coworkers, we created four combined BMI-fitness categories (20) allowing the first to fourth run-time quintile to represent “fit” and the fifth quintile to represent “unfit” participants. A BMI cutoff of 23.0 kg/m^2^ that reflected the threshold between the “acceptable risk” and “increased risk” categories in the Asian populations was used to distinguish between “BMI<23” and “BMI≥23” participants. The “BMI<23-fit” category served as the reference in subsequent analyses.

### Sensitivity analyses

Where appropriate, we conducted sensitivity analyses to review the robustness of our findings. First, after considering previous evidence of a J-shaped association between BMI and study outcomes (27), we ran additional regression analyses that controlled BMI as a categorical confounding factor. Second, we defined more granular BMI-fitness categories to assess how the selection of cutoff values would affect our estimates. Finally, to address the scenario where the proportional hazards assumption was inadequate, we identified the variable that drove the inadequacy and ran stratified Cox regression models that allowed the strata formed from the identified variable to have different baseline hazards.

MS Excel 2016 (Microsoft Corporation) and STATA Version 13 (StataCorp LLC) were used to conduct all statistical analyses. Findings with a *p*-value of <0.05 were considered statistically significant. We used the STROBE statement checklist (28) to ensure the completeness of our report.

### Conduct and oversight

All authors except the third author conceptualized the study while the sixth author directed the analytical methodology. The first, second, third, and sixth authors planned, executed, and verified the analyses. The original draft was reviewed and edited by all co-authors before submitting for publication. The authors have declared competing interests as listed at the end of this manuscript. No additional funding was required to conduct this study. Any sharing of original data is subject to the approval of the aforementioned data owners. Patients and the public were not involved in the design or conduct of this study. The study protocol was approved by the DSO National Laboratories—Singapore Armed Forces Institutional Review Board, Reference Number 0021/2019.

## Results

The final study sample comprised 212,631 healthy male participants who underwent medical examination between 01 January 1998 and 31 December 2015 and had no significant medical history that would have precluded them from participating in maximal intensity physical fitness testing.

At the time of medical examination, the mean age of participants was 19.1 years (standard deviation [SD] 1.4), the mean height was 1.72 m (SD 0.06), and the mean BMI was 21.7 kg/m^2^ (SD 3.9). The mean 2.4 km run-time recorded at the first fitness test was 678 s (SD 116). Further participant characteristics within each exposure category are shown in Tables 1, 2 and Supplementary Table 1. There was a positive relationship between BMI and the 2.4-km run-time with a Spearman correlation coefficient of 0.223. With 106,395 (50%) of participants entering the study period on 01 January 2007 and the rest entering between 02 January 2007 and 31 December 2015, the mean age at the year of entry into the study period was 22.4 years (SD 3.3). Primary analyses at the time of the first MACE comprised 2,043,278 person-years of follow-up with each participant contributing on average 9.6 (SD 2.7) years. Secondary analyses of time until ACM comprised 2,044,269 person-years. Of the 371 participants experiencing the first MACE, 176 (47%) participants recorded the first AMI event, 136 (37%) participants recorded the first acute stroke event, 59 (16%) underwent the first PCI procedure, 5 (1%) underwent the first coronary artery bypass grafting procedure, and 48 (13%) died of CV causes. This translated to an incidence rate of 1.82 first MACE per 10,000 person-years. The average age at the time of the first MACE was 33.4 years (SD 4.9). There were 243 deaths due to all causes with 195 (80%) categorized as “non-CV death” or “missing causes of death.” This translated to an incidence rate of 1.19 deaths due to all causes per 10,000 person-years. The average age at the time of death was 31.0 years (SD 5.5). Detailed statistics for each run-time quintile and BMI category can be found in the Supplementary Tables 2, 3.

**Table 1:** Characteristics of the participants by run-time quintile (*n* = 212,631).

	**Run time quintile**					
	**Q1**	**Q2**	**Q3**	**Q4**	**Q5**	**Total**
*n* (%)	42,611 (20.0)	42,739 (20.1)	42,244 (19.9)	42,797 (20.1)	42,240 (19.9)	212,631 (100)
**Run-time in s**						
Range	451 to 585	586 to 643	644 to 676	677 to 725	726 to 1,800	451 to 1,800
**Age in years at BMI measurement**						
Mean (SD)	18.9 (1.2)	19.0 (1.3)	19.1 (1.3)	19.2 (1.4)	19.4 (1.5)	19.1 (1.4)
**Height in m**						
Mean (SD)	1.72 (0.06)	1.72 (0.06)	1.72 (0.06)	1.72 (0.06)	1.72 (0.06)	1.72 (0.06)
**BMI in kg/m** ^**2**^						
Mean (SD)	20.6 (2.5)	21.0 (3.0)	21.3 (3.4)	21.8 (3.8)	24.0 (5.3)	21.7 (3.9)
**Age at 1st Test in years**						
Mean (SD)	20.2 (1.4)	20.4 (1.5)	20.5 (1.5)	20.9 (1.8)	21.5 (2.2)	20.7 (1.8)

BMI, Body mass index.

Q—Quintile.

SD—Standard deviation.

**Table 2:** Frequency counts and column percentages for run-time quintiles and BMI category at baseline (*n* = 212,631).

	**Run time quintile**					
	**Q1**	**Q2**	**Q3**	**Q4**	**Q5**	**Total**
WHO BMI cutoffs for Asians in kg/m^2^	***n*** **(%)**	***n*** **(%)**	***n*** **(%)**	***n*** **(%)**	***n*** **(%)**	***n*** **(%)**
“Underweight” ≤ 18.4	7,780 (18)	8,337 (20)	8,011 (19)	7,735 (18)	5,546 (13)	37,409 (18)
“Acceptable risk” 18.5 to 22.9	28,068 (66)	24,989 (58)	22,795 (54)	21,324 (50)	15,653 (37)	112,829 (53)
“Increased risk” 23.0 to 27.4	6,244 (15)	8,056 (19)	9,310 (22)	10,271 (24)	10,725 (25)	44,606 (21)
“High risk” ≥27.5	519 (1)	1,357 (3)	2,128 (5)	3,467 (8)	10,316 (24)	17,787 (8)
**Total**	42,611 (100)	42,739 (100)	42,244 (100)	42,797 (100)	42,240 (100)	212,631 (100)

BMI, Body mass index; WHO, World Health Organization; Q, quintile.

### Cardiorespiratory fitness

**Figure 2:**
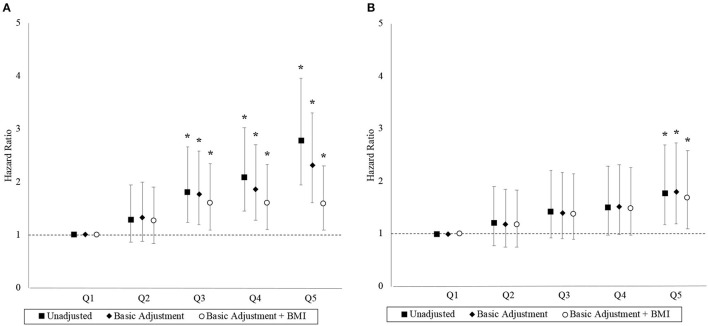
Hazard ratios with 95% confidence interval by run-time quintile. Panels **(A, B)** correspond to the MACE and ACM outcomes, respectively. Basic adjustments also included the year of BMI measurement and age at time of entry into the study period as predictors in the models. The models were further adjusted for BMI. An asterisk (*) denotes a hazard ratio that is significantly different from 1 (i.e., *p* < 0.05).

The association between the 2.4-km run-time and hazard for MACE and ACM are depicted by the hazard ratios (HRs) in Figure 2. Each successive run-time quintile had an incrementally higher unadjusted hazard for MACE. Compared to the first run-time quintile, final adjusted HRs for MACE were 1.26 (95% CI 0.84–1.91), 1.60 (95% CI 1.09–2.35), 1.60 (95% CI 1.10–2.33), and 1.58 (95% CI 1.09–2.30) in the second to fifth quintiles, respectively. The linear trend was nonsignificant (*p* = 0.188) with the HRs plateauing from the third quintile, while the run-time quintile as a categorical variable was a significant factor (*p* < 0.001), suggesting a non-linear trend. The proportional hazard assumption was adequate in the final model for MACE (*p* = 0.179).

Similarly, each successive run-time quintile was associated with an incrementally higher unadjusted hazard for ACM although the associations were generally weaker. Compared to the first run-time quintile, final adjusted HRs for ACM were 1.17 (95% CI 0.74–1.84), 1.38 (95% CI 0.89–2.14), 1.48 (95% CI 0.97–2.27), and 1.68 (95% CI 1.09–2.59) in the second to fifth quintiles, respectively. The linear trend was significant (*p* = 0.005) and the proportional hazard assumption was adequate in the final model for ACM (*p* = 0.057).

### Body mass index

**Figure 3:**
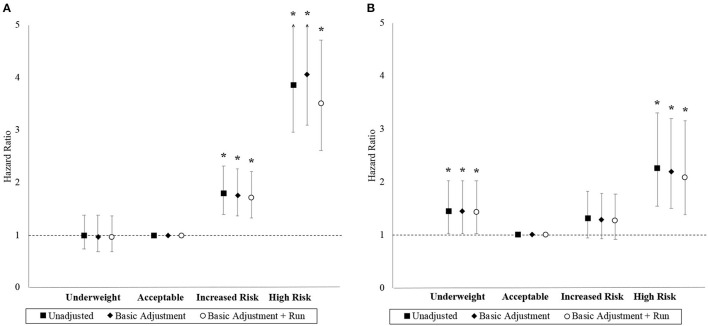
Hazard ratios with 95% confidence interval by BMI category. Panels **(A, B)** correspond to the MACE and ACM outcomes, respectively. Basic adjustments also included the year of BMI measurement and age at time of entry into the study period as predictors in the models. Models were further adjusted for the 2.4 km run-time. An asterisk (*) denotes a hazard ratio that is significantly different from 1 (i.e., *p* < 0.05).

The association between BMI and hazard for MACE and ACM are depicted in Figure 3. The “increased risk” and “high risk” BMI categories have higher unadjusted hazards for MACE than the “acceptable risk” BMI category. Compared to the “acceptable risk” BMI category, the final adjusted HRs for MACE were 0.97 (95% CI 0.69–1.37), 1.71 (95% CI 1.33–2.21), and 3.51 (95% CI 2.61–4.72) in the “underweight,” “increased risk,” and “high risk” BMI categories, respectively. The linear trend was significant (*p* = 0.047), and the proportional hazard assumption was adequate in the final model for MACE (*p* = 0.314).

The “underweight” and “high risk” BMI categories had higher unadjusted hazards for ACM than the “acceptable risk” BMI category, but both the “acceptable risk” and “increased risk” BMI categories had comparable unadjusted hazards. Compared to the “acceptable risk” BMI category, the final adjusted HRs for ACM were 1.43 (95% CI 1.02–2.02), 1.27 (95% CI 0.92–1.77), and 2.08 (95% CI 1.38–3.16) in the “underweight,” “increased risk,” and “high risk” BMI categories, respectively. Although the linear trend was non-significant (*p* = 0.278), BMI as a categorical variable was a significant factor (*p* = 0.024) with a J-shaped trend observed. The proportional hazards assumption was inadequate in the final model for ACM (p=0.044). Stratified Cox models with the strata corresponding to the “age at time of entry into study period” being categorized by its quantiles resolved the non-proportional hazards problem with minimal impact on effect estimates.

### Combined associations of cardiorespiratory fitness and body mass index

**Figure 4:**
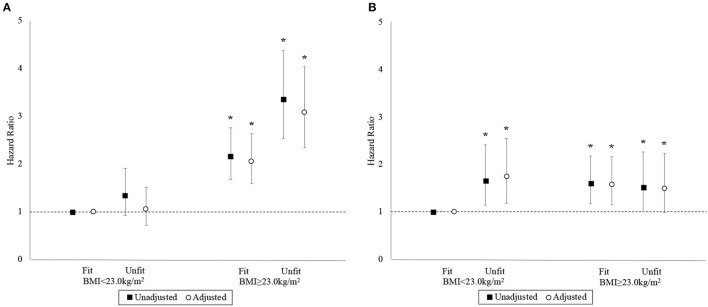
Hazard ratios with 95% confidence interval by BMI-fitness category. Panels **(A, B)** correspond to the MACE and ACM outcomes, respectively. Basic adjustments also included the year of BMI measurement and age at time of entry into the study period as predictors in the models. An asterisk (*) denotes a hazard ratio that is significantly different from 1 (i.e., *p* < 0.05).

The associations between the BMI-fitness categories and the hazards for MACE and ACM are depicted in Figure 4. The “BMI<23-unfit” category and the “BMI<23-fit” reference category had similar unadjusted hazards for MACE, while those in the “BMI≥23-fit” and “BMI≥23-unfit” categories experienced markedly greater hazards than the reference category. Compared to the “BMI<23-fit” reference category, final adjusted HRs for MACE were 1.05 (95% CI 0.72–1.51), 2.06 (95% CI 1.60–2.64), and 3.08 (95% CI 2.35–4.04) for the “BMI<23-unfit,” “BMI≥23-fit,” and “BMI≥23-unfit categories, respectively. The proportional hazard assumption was adequate in the final model for MACE (*p* = 0.526).

Compared to the “BMI<23-fit” reference category, the final adjusted HRs for ACM were 1.74 (95% CI 1.18–2.55), 1.58 (95% CI 1.16–2.16), and 1.49 (95% CI 0.99–2.24) for “BMI<23-unfit,” “BMI≥23-fit,” and “BMI≥23-unfit,” respectively. The proportional hazards assumption was inadequate in the final model for ACM (*p* = 0.029). Yet again, stratified Cox models resolved the non-proportional hazards problem with the same variable identified earlier and minimal impact on effect estimates.

### Sensitivity analyses

In the model that controlled for BMI as a categorical variable (Supplementary Figure 1), the effect estimates were largely consistent with the original model for MACE. In the model for ACM, however, the lower bound of the 95% confidence in the fifth run-time quintile dipped to below one, although the test for the trend remained significant (*p* = 0.018).

We created six new BMI-fitness categories comprising three fitness levels along with our original BMI cutoff of 23.0 kg/m^2^. “High fitness” comprised the first and second run-time quintiles and “BMI<23-high fitness” served as the new reference category. “Moderate fitness” comprised the third and fourth quintiles, while “low fitness” comprised only the fifth quintile. Crude incidence rates within each category (Supplementary Tables 4, 5) and the Cox model output (Supplementary Figure 2) suggested that the relationships were largely consistent with the output from models that used four BMI-fitness categories.

Among our final adjusted Cox models for ACM, two of the models did not satisfy the proportional hazards assumption. Age at the time of entry into the study period was identified as the variable of concern, and the nonproportional hazards problem was addressed by using models that stratified on this variable (i.e., stratified Cox models). Since differences in HRs were marginal, we reported the results from the Cox model. All final adjusted models for MACE satisfied the proportional hazards assumption.

## Discussion

### Summary of key findings

This study examined the associations of 2.4-km run-times and BMI with CV morbidity and mortality outcomes in healthy young men. Longer 2.4-km run-times, hence, lower estimated CRF, and higher BMI were associated with an increased hazard of MACE. There was also a significant dose–response effect for the 2.4-km run-time on the hazard of ACM. Combined analyses showed that the membership in the “BMI≥23-fit” category attenuated but did not eliminate the increased risk of MACE associated with increased BMI.

### Cardiorespiratory fitness as a predictor of MACE and ACM

Our study has served to further substantiate the findings from studies that described an inverse relationship between CRF and the biomarkers of CVD among adolescents and young adults (13–16). The study was also designed to emulate key studies conducted in older adults (7) that had established CRF as a predictor of MACE and ACM incidence. Primary analyses showed that CVD hazards were elevated 1.60 times from the third run-time quintile, a finding that was consistent with the risk associations observed in a Swedish conscription cohort (20). While the aforementioned cohort produced a linear dose–response curve for the association between CRF and CVD risk, we suspect that differences in study outcomes and follow-up times could explain the absence of the said effect in our sample. Secondary analyses showed that the ACM hazards were elevated 1.68 times in the fifth run-time quintile, an effect estimate that was similar to the previous meta-analysis findings (7).

### BMI as a predictor of MACE and ACM

Our analyses reiterate previous findings that elevated BMI in adolescence (11, 12) and early adulthood (29) increase the risks of CVD and ACM. Moreover, the proportion of participants who were classified as having “increased risk” or “high risk” BMI and were shown to experience higher hazards of MACE was 29% in our study. In the Swedish conscription cohort, (20) the proportion of participants who were classified as “overweight” or “obese,” i.e., BMI≥25.0 kg/m^2^ according to the WHO classification (25) and were shown to experience greater hazards of CVD was 10%. While this difference in proportions was substantial, it is important to note that the Swedish sample was recruited from 1972 to 1994, when global obesity prevalence had only just begun to accelerate (10). Secondary analyses showed elevated hazards of ACM at the upper and lower extremes of the BMI spectrum, a finding that was consistent with the risk associations reported in an Israeli cohort of military conscripts (30). Our study, therefore, strongly reinforces the notion that ACM risks exhibit a J-shaped distribution in relation to BMI even among healthy young adults (27, 31).

### Combined associations of cardiorespiratory fitness and BMI

Previous studies in adolescents (15) and older adults (4) have described how high CRF could fully compensate for the negative effects of elevated BMI. Our primary analyses demonstrated an attenuating effect of shorter run-times on MACE risks associated with elevated BMI that has been described in an older cohort (32). Secondary analyses did not show any attenuating effect of shorter run-times on ACM risks associated with elevated BMI. A relatively short duration of follow-up might explain these discrepancies with the literature (33). On the opposite end of the spectrum, however, our finding that run-times, hence estimated CRF, could differentiate ACM risk among participants with a BMI of <23.0 kg/m^2^ might offer additional insights into the early health hazards associated with a low BMI (31).

### Implications

Under primary care settings, the 10-year CVD risk estimation is a key component of CVD prevention for adults aged 40 to 75 years (34). Our study suggests that measures of BMI and CRF could potentially be used in CVD risk estimation in men under the age of 40 years. Moreover, where monitoring and surveillance data for CRF are available (35, 36), our findings could aid in determining key thresholds for public health action. Finally, our findings on the combined effects of estimated CRF and BMI offer a degree of nuance to the discussion on the “fat-but-fit” paradigm (19) to suggest that superior fitness might not fully eliminate the adverse effects of elevated body weight in young men.

### Strengths

To the best of our knowledge, this is the first large-scale longitudinal study examining BMI, estimated CRF, and the longitudinal risk of CVD events and ACM in young Asian men. Our strict eligibility criteria produced a relatively homogeneous sample that had been in good health at baseline, thus assuring the temporality between exposures and outcomes. Our reliance on national registry data, where the reports of death and AMI events are required by law, also increased the quality of outcome measures.

### Limitations

A large number of potential participants were excluded on the basis of missing data, thus limiting the representativeness of the sample. Furthermore, most studies cited in our discussion used criterion-measure laboratory protocols involving either a treadmill or cycle ergometer to assess CRF (37). While a meta-analysis of 123 studies based on the criterion-validity of field-based test protocols clearly favors the modified Cooper's test (38), we must consider how misclassification in our exposure data might have attenuated the effect estimates in our population. Our study population also comprised healthy male military conscripts of Asian ethnicity, meaning that comparisons with other populations need to account for putatively greater CRF, lower BMI, and Asian BMI cutoff values in our sample.

Concerning the MACE outcomes, the registry data were consistently available from 01 January 2007, and information on losses to follow-up, for example, through migration, were lacking. The resulting undercount of MACE and inflated follow-up time indicate that we have likely underestimated the MACE incidence for our cohort. Finally, important demographic factors, such as ethnicity, education, socioeconomic status, and smoking, and clinical factors, such as blood pressure, serum lipid concentration, and fasting glucose levels, were not captured in this study limiting our ability to account for important confounders of the relationships between BMI, CRF, MACE, and ACM. This implies that our findings might not be applicable in the context of individual risk management but are potentially more relevant to public health promotion.

## Conclusion

While previous research on adolescent males and young men focused on the relationship between CRF, BMI, and cardiometabolic risk factors, our analyses examined the incidence of hard CVD outcomes and ACM within a relatively short duration of follow-up. Our study showed that a greater estimated CRF did not fully mitigate the hazards associated with elevated BMI, thus weakening the argument of a “fat-but-fit” paradigm in young men. Therefore, this study suggests that public health messaging strategies targeting adolescent males and young men ought to address CRF and BMI concurrently.

## Evidence before this study

The “fat-but-fit” paradigm, which is now commonplace in health promotion, indicates that high cardiorespiratory fitness (CRF) mitigates the harmful effects of high body mass index (BMI). This phenomenon is largely underpinned by evidence from older populations.

## Added value of this study

In our study, young Asian men entering the National Service in Singapore demonstrated that CRF and BMI in adolescence and early adulthood are powerful determinants of cardiovascular disease and all-cause mortality. While a high CRF did not completely mitigate the risks associated with high BMI, a strong attenuating effect was demonstrable within a relatively short duration of follow-up.

## Implications of all available evidence

Our findings reiterate the need for population-wide physical activity and CRF promotion that is concurrent with obesity prevention in adolescence and early adulthood.

## Data availability statement

The original contributions presented in the study are included in the article/Supplementary material, further inquiries can be directed to the corresponding author.

## Ethics statement

The study protocol which involved human participants was reviewed and approved by DSO National Laboratories - Singapore Armed Forces Institutional Review Board, Reference Number 0021/2019. The Ethics Committee waived the requirement of written informed consent for participation.

## Author contributions

AG, JY, WC, KY, and FM-R conceptualized the study while CT directed the analytical methodology. AG, JY, JN, and CT planned, executed, and verified the analyses. The original draft was reviewed and edited by all co-authors before submitting for publication.
