# Supplementary material for: Cardiorespiratory fitness, body mass index, cardiovascular disease, and mortality in young men: A cohort study
Source: Front Public Health. 2023 Feb 15;11:1076065. doi: 10.3389/fpubh.2023.1076065 (PMC9975166; doi:10.3389/fpubh.2023.1076065)
Supplement: Supplementary file 1 [file Data_Sheet_1.docx]

**Supplementary Material**

**Supplementary Table S1.** Characteristics of the participants by BMI-fitness categories (n=212,631)

|  |  |  |  |  |  |
| --- | --- | --- | --- | --- | --- |
|  | BMI -Fitness Category | | | |  |
|  | **BMI<23-fit** | **BMI<23-unfit** | **BMI≥23-fit** | **BMI≥23-unfit** | **Total** |
| n (%) | 129,039 | 21,199 | 41,352 | 21,041 | 212,631 (100) |
| **Run time in s** |  |  |  |  |  |
| Range | 451 to 725 | 726 to 1796 | 462 to 725 | 726 to 1,800 | 451 to 1,800 |
| Mean (SD) | 630 (53) | 827 (115) | 648 (50) | 878 (147) | 678 (115) |
| **Age in years at BMI measurement** |  |  |  |  |  |
| Mean (SD) | 19.0 (1.3) | 19.4 (1.6) | 19.3 (1.4) | 19.3 (1.5) | 19.1 (1.4) |
| **Height in m** |  |  |  |  |  |
| Mean (SD) | 1.72 (0.06) | 1.71 (0.06) | 1.72 (0.06) | 1.72 (0.06) | 1.72 (0.06) |
| **BMI in kg/m^2^** |  |  |  |  |  |
| Mean (SD) | 19.8 (1.8) | 19.8 (2.0) | 25.6 (2.6) | 28.2 (4.1) | 21.7 (3.9) |
| **Age at 1^st^ Test in years** |  |  |  |  |  |
| Mean (SD) | 20.4 (1.5) | 21.7 (2.3) | 20.7 (1.6) | 21.3 (2.1) | 20.7 (1.8) |

BMI – body-mass index

kg – kilogram

m – metre

Q – quintile

s – second

SD – standard deviation

**Supplementary Table S2**. First MACE, ACM, CV mortality, first AMI events and first stroke events by run-time quintiles

|  | Run-time Quintiles | | | | |  |
| --- | --- | --- | --- | --- | --- | --- |
|  | **Q1** | **Q2** | **Q3** | **Q4** | **Q5** | **Total** |
| n for primary outcome (MACE) | 42,611 | 42,739 | 42,244 | 42,797 | 42,240 | 212,631 |
|  |  |  |  |  |  |  |
| **First MACE events: n** | 41 | 51 | 72 | 90 | 117 | 371 |
| Mean age at event (SD) | 31.8 (5.7) | 32.5 (5.5) | 33.3 (5.2) | 33.9 (4.5) | 34.1 (4.2) | 33.4 (4.9) |
| Crude incidence rate* | 9.6 | 11.9 | 17.0 | 21.0 | 27.7 | 17.4 |
|  |  |  |  |  |  |  |
| **ACM events: n** | 35 | 41 | 48 | 55 | 64 | 243 |
| Mean age at death (SD) | 29.2 (4.8) | 29.2 (5.2) | 30.9 (5.6) | 31.7 (5.7) | 32.7 (5.7) | 31.0 (5.5) |
| Crude incidence rate* | 8.2 | 9.6 | 11.4 | 12.9 | 15.2 | 11.4 |
|  |  |  |  |  |  |  |
| **CV mortality events: n** | 6 | 6 | 6 | 12 | 18 | 48 |
| Mean age at death (SD) | 31.5 (4.8) | 29.3 (5.8) | 32.2 (5.3) | 34.9 (3.4) | 32.8 (4.9) | 33.4 (4.8) |
| Crude incidence rate* | 1.4 | 1.4 | 1.4 | 2.8 | 4.3 | 2.3 |
|  |  |  |  |  |  |  |
| **First AMI events: n** | 19 | 27 | 32 | 38 | 60 | 176 |
| Mean age at event (SD) | 35.3 (3.8) | 33.7 (5.3) | 34.2 (4.6) | 35.4 (3.6) | 34.4 (4.0) | 34.6 (4.2) |
| Crude incidence rate* | 4.5 | 6.3 | 7.6 | 8.9 | 14.2 | 8.3 |
|  |  |  |  |  |  |  |
| **First stroke events: n** | 15 | 15 | 32 | 35 | 39 | 136 |
| Mean age at event (SD) | 26.9 (4.5) | 30.3 (5.1) | 32.1 (5.5) | 32.1 (5.3) | 34.2 (4.0) | 31.9 (5.3) |
| Crude incidence rate* | 3.5 | 3.5 | 7.6 | 8.2 | 9.2 | 6.4 |

* per 10,000 participants

ACM – all-cause mortality

AMI – acute myocardial infarction

CV – cardiovascular

MACE – major acute cardiovascular event

Q – quintile

SD – standard deviation

**Supplementary Table S3**. First MACE, ACM, CV mortality, first AMI events and first stroke events by BMI categories

| BMI Category for Asian Populations | ‘under-weight’ | ‘acceptable risk’ | ‘increased risk’ | ‘high risk’ |  |
| --- | --- | --- | --- | --- | --- |
| BMI in kg/m^2^ | ≤18.4 | 18.5 to 22.9 | 23.0 to 27.4 | ≥27.5 | **Total** |
| n for primary outcome (MACE) | 37,409 | 112,829 | 44,606 | 17,787 | 212,631 |
|  |  |  |  |  |  |
| **First MACE events: n** | 41 | 145 | 101 | 84 | 371 |
| Mean age at event (SD) | 32.4 (5.1) | 33.2 (5.2) | 33.6 (4.9) | 34.1 (4.2) | 33.4 (4.9) |
| Crude incidence rate* | 11.0 | 12.9 | 22.6 | 47.2 | 17.4 |
|  |  |  |  |  |  |
| **ACM events: n** | 48 | 105 | 54 | 36 | 243 |
| Mean age at death (SD) | 29.1 (4.7) | 30.3 (5.6) | 32.6 (5.7) | 32.8 (5.5) | 31.0 (5.5) |
| Crude incidence rate* | 12.8 | 9.3 | 12.1 | 20.2 | 11.4 |
|  |  |  |  |  |  |
| **CV mortality events: n** | 8 | 17 | 14 | 9 | 48 |
| Mean age at death (SD) | 30.9 (4.4) | 33.2 (3.9) | 31.9 (6.5) | 34.2 (3.9) | 33.4 (4.8) |
| Crude incidence rate* | 2.1 | 1.5 | 3.1 | 5.1 | 2.3 |
|  |  |  |  |  |  |
| **First AMI events: n** | 11 | 67 | 58 | 40 | 176 |
| Mean age at event (SD) | 35.6 (4.6) | 35.0 (4.1) | 34.3 (4.6) | 34.0 (3.8) | 34.6 (4.2) |
| Crude incidence rate* | 2.9 | 5.9 | 13.0 | 22.5 | 8.3 |
|  |  |  |  |  |  |
| **First stroke events: n** | 23 | 58 | 26 | 29 | 136 |
| Mean age at event (SD) | 31.3 (4.9) | 31.1 (6.0) | 32.6 (4.2) | 33.6 (4.6) | 31.9 (5.3) |
| Crude incidence rate* | 6.1 | 5.1 | 5.8 | 16.3 | 6.4 |

* per 10,000 participants

ACM – all-cause mortality

AMI – acute myocardial infarction

CV – cardiovascular

kg - kilogramme

MACE – major acute cardiovascular event

s - second

SD – standard deviation

**Supplementary Table S4.** Cross-tabulation of run-time and BMI category baseline (n=212,631) with number of first MACE recorded during study period.

|  |  | **Run-time Quintile** | | | | |  |
| --- | --- | --- | --- | --- | --- | --- | --- |
|  |  | **Q1** | **Q2** | **Q3** | **Q4** | **Q5** | **Total** |
| WHO BMI Category for Asian Populations |  |  |  |  |  |  |  |
| ‘underweight’ | MACE events: n | 5 | 7 | 12 | 12 | 5 | **41** |
| BMI ≤18.5 | Population: n | 7,780 | 8,337 | 8,011 | 7,735 | 5,546 | **37,409** |
| ‘acceptable risk’ | MACE events: n | 28 | 24 | 29 | 33 | 30 | **145** |
| BMI 18.5 to 22.9 | Population: n | 28,068 | 24,989 | 22,795 | 21,324 | 15,653 | **112,829** |
| ‘increased risk’ | MACE events: n | 8 | 17 | 23 | 22 | 31 | **101** |
| 23.0 to 27.4 | Population: n | 6,244 | 8,056 | 9,310 | 10,271 | 10,725 | **44,606** |
| ‘high risk’ | MACE events: n | 0 | 3 | 8 | 23 | 50 | **84** |
| ≥27.5 | Population: n | 519 | 1,357 | 2,128 | 3,467 | 10,316 | **17,787** |
| **Total** | MACE events: n | **41** | **51** | **72** | **90** | **117** | **371** |
|  | Population: n | 42,611 | 42,739 | 42,244 | 42,797 | 42,240 | 212,631 |

BMI – body-mass index

MACE – major acute cardiovascular event

Q – quintile

**Supplementary Table S5.** Cross-tabulation of run-time quintile and BMI category at baseline (n=212,631) with number of ACM events recorded during study period.

|  |  | **Run-time Quintiles** | | | | |  |
| --- | --- | --- | --- | --- | --- | --- | --- |
|  |  | **Q1** | **Q2** | **Q3** | **Q4** | **Q5** | **Total** |
| WHO BMI Category for Asian Populations |  |  |  |  |  |  |  |
| ‘underweight’ | ACM events: n | 7 | 7 | 8 | 16 | 10 | **48** |
| BMI ≤18.5 | Population: n | 7,780 | 8,337 | 8,011 | 7,735 | 5,546 | **37,409** |
| ‘acceptable risk’ | ACM events: n | 19 | 21 | 23 | 17 | 25 | **105** |
| BMI 18.5 to 22.9 | Population: n | 28,068 | 24,989 | 22,795 | 21,324 | 15,653 | **112,829** |
| ‘increased risk’ | ACM events: n | 7 | 11 | 11 | 15 | 10 | **54** |
| 23.0 to 27.4 | Population: n | 6,244 | 8,056 | 9,310 | 10,271 | 10,725 | **44,606** |
| ‘high risk’ | ACM event: n | 2 | 2 | 6 | 7 | 19 | **36** |
| ≥27.5 | Population: n | 519 | 1,357 | 2,128 | 3,467 | 10,316 | **17,787** |
| **Total** | **ACM events: n** | 35 | 41 | 48 | 55 | 64 | 243 |
|  | **Population: n** | 42,611 | 42,739 | 42,244 | 42,797 | 42,240 | 212,631 |

ACM – all-cause mortality

BMI – body-mass index

Q – quintile

**Supplementary Figure 1. Hazard Ratios with 95% Confidence Interval by Run-time Quintile. Panel A and B correspond to MACE and ACM outcomes respectively.** Basic adjustments also included year of BMI measurement and age at time of entry into study as predictors in the models. Models were further adjusted for BMI and BMI category as shown. An asterisk (*) denotes a hazard ratio that is significantly different from 1 (i.e., p<0.05).

ACM – all-cause mortality

BMI – body-mass index

MACE – major acute cardiovascular event

Q – quintile

**Supplementary Figure 2. Hazard Ratios with 95% Confidence Interval by BMI-fitness Category. Panel A and B correspond to MACE and ACM outcomes respectively.** Adjustments also included year of BMI measurement and age at time of entry into study as predictors in the models. An asterisk (*) denotes a hazard ratio that is significantly different from 1 (i.e., p<0.05).

ACM – all-cause mortality

BMI – body-mass index

MACE – major acute cardiovascular event
